# A sustainable solution to address the unmet need for specialist and general surgical services of children in under-resourced contexts

**DOI:** 10.11604/pamj.2023.45.180.41361

**Published:** 2023-08-24

**Authors:** Diaaeldinn Yaseen Salman, Boniphace Tresphory, Pierluigi Lelli Chiesa, Alessandro Calisti

**Affiliations:** 1 Gezira National Centre for Pediatric Surgery, Wad Madani, Sudan,; 2 Department of Surgery, Consolata Hospital Ikonda, Njombe, Tanzania,; 3 Department of Pediatric Surgery, University of Chieti Pescara, Chieti, Italy,; 4 Department of Pediatric Surgery, San Camillo Forlanini Hospital, Roma, Italy

**Keywords:** Children, surgery, low resource

## To the editors of the Pan African Medical Journal

The shortage of surgical services for children affects low resources contexts (LRC) of sub-Saharan Africa, where the proportion of specialists is 1 to 6,000,000 for a population of 45% under 14 years of age, compared with one pediatric surgeon for every 100,000 children, 0-15 years of age of Western countries [1]. In far extra-urban and rural areas, lack of transportation and poverty hamper access to the few specialist facilities. Neonates with correctable congenital disabilities or children with life-threatening conditions may die before being seen, and the real burden of pediatric surgical diseases may be underestimated [2]. Although more investments in human resources and child health facilities are recommended, full coverage for the burden of pediatric congenital and acquired surgical diseases cannot be a short-term achievement.

General surgeons in district and rural hospitals usually treat all children outside specialist centers, but the quality of care may sometimes be far below the recommended standard [3]. Still, generally, they cannot guarantee appropriate management for rare and complex conditions like congenital abnormalities, tumors, and severe injuries. Neonatal anorectal malformations are the most commonly recorded (57%-67%) and treated abnormalities (up to 64%). In a non-specialist context, even a limited procedure, like an emergency colostomy, frequently does not follow current guidelines and risks compromising further management, as we documented [4].

For a broad sector of paediatric surgery, the same skills and facilities needed for major conditions are not required [5]. A workforce of general surgeons interested in children's surgery and, with additional training, acting in under-resourced areas within a “Hub and Spoke” model (H&SM) of care delivery, also adopted in some European countries [6], is a realistic solution to deal with the load of the so-called general pediatric surgery [7]. Herniotomies, orchidopexies, distal urethroplasty, pyloromyotomy, urinary diversions, colostomies, and other accessible gastrointestinal surgeries can be appropriately managed locally (Spoke), benefiting families and patients in the area and reducing costs. The few highly equipped centers (Hub), attracting enough workload to be reliable and sustainable, will deal with more complex cases like neonatal cases, oncology, urology, hepatobiliary disease, and major trauma referred from Spoke according to shared guidelines and protocols.

Our opinion is enforced by recent experience (2016 to date) in two East African contexts. In Sudan, the Gezira National Centre for Pediatric Surgery acts as the Hub for the highly populated state of Gezira [8]. In Tanzania, the Consolata Hospital Ikonda, a regional hospital, acts as a spoke for the district of Makete [9]. Two training levels for surgical residents are needed according to an Indian experience [10]. Surgeons going to practice in peripheral health facilities [Spoke] need basic paediatric knowledge and practical training (level-one - General Paediatric Surgery) with at least six months of coaching provided in a Specialist Paediatric Surgical Unit to be completed under the tutorship of an experienced general paediatric surgeon in a District General Hospital. Surgeons for tertiary centers (Hub) should follow a level-two program with the higher approved surgical training program in specialist paediatric surgery provided at qualified centers.

Spoke centres need a stable paediatric surgical workload from their catchment areas and some anaesthesia and resuscitation expertise. A close link should exist with the Specialist Paediatric Hub (also by telemedicine portals) to share decision-making algorithms and management protocols for early-stage more complex diseases [7]. Service goals will also include: i) a Spoke clinical lead for paediatric cases; ii) a Spoke anaesthetists committed to good quality elective and emergency service; iii) periodic visiting specialist surgeon (outreach surgery) from Hub Centre; iv) Hub and Spoke multidisciplinary clinical meetings and audits on outcomes.

An H&SM is implemented through a staged approach. In the first stage, medical and nursing staff from the Hub will visit the Spoke. An elective list of cases should maintain local staff's skills, and surgeries should be arranged weekly or monthly. As a final step, spoke general surgeons can deal autonomously with children's general surgery, have their outpatients' surgical clinics, provide appropriate early management to particular cases, and refer them following well-established guidelines. A continuing education and supervision program will interest hub and spoke paediatric surgical services.

## Conclusion

More resources and a new approach are required to reduce the dramatic imbalance in access to paediatric surgical services between children living in high and low-resource contexts. The risk of undue treatment may threaten for a long time to come thousands of patients needing surgery and without access to specialist health facilities. Upgrading peripheral hospitals to deliver appropriate general paediatric surgical treatment and early management of more complex cases before referring to a specialist tertiary centre is a possible sustainable solution. A better-trained Health workforce at the rural/district level within an established H&SM network can play a significant role. Although a controlled and decentred model has already been recommended for specialist health delivery in Africa, it has yet to be developed as it should be desirable [1].
